# High-fat diet from parental generation exaggerates body and adipose tissue weights in pregnant offspring

**DOI:** 10.1371/journal.pone.0237708

**Published:** 2020-08-20

**Authors:** Frank T. Spradley

**Affiliations:** Department of Surgery, University of Mississippi Medical Center, Jackson, MS, United States of America; Royal College of Surgeons in Ireland, IRELAND

## Abstract

Parental high-fat diet (HFD) programs for obesity and hypertension in female offspring in rats, but it is unknown how the pregnancies of these offspring are impacted. Therefore, the hypothesis was tested that parental HFD exaggerates obesity and hypertension during pregnancy of the offspring. Wistar Hannover rat dams (the parental, P generation) were maintained on normal-fat diet (NFD) or HFD from weaning and were kept on respective diets through pregnancy and lactation. Their offspring (the first filial, F1 generation) were weaned onto the same diet as the P generation, or they were changed to the other diet to determine if combined HFD in the P and F1 generations exaggerates body weight and blood pressure levels during pregnancy in these offspring. This diet paradigm resulted in the following groups of pregnant F1 offspring: P-NFD/F1-NFD, P-HFD/F1-NFD, P-NFD/F1-HFD, and P-HFD/F1-HFD. Maternal body and adipose tissue weights were greatest in the P-HFD/F1-HFD group compared to the other 3 groups by the end of pregnancy. Plasma leptin and conscious mean arterial blood pressure were not significantly different between any group, although there was a main effect for increased blood pressure in the F1-HFD groups. Circulating levels of the antihypertensive pregnancy factor, placental growth factor (PlGF), were assessed. Although average PlGF levels were similar among all groups, correlative studies revealed that lower levels of PlGF were associated with higher blood pressure only in the P-HFD/F1-HFD group. In summary, HFD feeding from the P generation exaggerated HFD-induced body and adipose tissue weights in the pregnant offspring.

## Introduction

Increasing evidence supports that insults in the parental (P) generation, including during the perinatal period of gestation and lactation, have lasting effects on body weight and blood pressure regulation in female offspring; this has been reported in several studies in rodents. It was found that a high-fat diet (HFD) started at 3 weeks of age and maintained throughout pregnancy and lactation in the P generation resulted in offspring of the first filial (F1) generation having increased body and adipose tissue (fat) weights along with elevations in blood pressure [1, 2]. Another study showed that the combination of HFD feeding in the P generation and from weaning in the F1 generation resulted in exaggerated body and fat weights in these offspring, but not exaggerated blood pressure levels [3, 4]. These data support that HFD during the P generation predisposes to exaggerated HFD-induced body fat weights in the female offspring accompanied by elevated blood pressure.

Although the above investigations showed that HFD in the P generation affects body weight and blood pressure regulation in the F1 generation, those experiments were conducted in non-pregnant female offspring. Therefore, it is unknown how HFD consumption in the P generation impacts the pregnancies of the F1 generation. What has been studied are the consequences of HFD on pregnancies in the P generation of dams. We previously showed that HFD feeding from early age and continued through pregnancy resulted in a slight increase in fat weight and elevated blood pressure by the end of pregnancy at gestational day 19 in rats [5]. Others have also reported that HFD promotes increased fat weight in the P generation during their pregnancies [6–9]. However, it has not been examined whether HFD feeding during pregnancy in the P generation alters body or fat weights as well as blood pressure in the F1 offspring during their own pregnancies. Moreover, it is unknown whether combined HFD feeding in the P and F1 generations would exaggerate body fat or blood pressure levels during pregnancy of the F1 offspring. It is timely to assess whether there are multigenerational effects of HFD on pregnancy because of the increasing prevalence of overweight or obesity occurring in pregnancies around the world [10]. Epidemiologists have reported that increased maternal body weight in the P generation is associated with the development of obesity in their offspring [11]. However, little is known about body weight and adipose tissue mass during pregnancy in these offspring, which highlights the novelty of the current study.

In this study, it was examined whether HFD compared to normal-fat diet (NFD) in the P generation promotes obesity and hypertension in the F1 offspring during pregnancy in the experimental groups named, P-NFD/F1-NFD and P-HFD/F1-NFD. It was also examined whether a combination of HFD feeding during the P and F1 generations exaggerates obesity and hypertension in the pregnant F1 offspring using the experimental groups named, P-NFD/HFD and P-HFD/F1-HFD. Furthermore, circulating levels of the pregnancy factor, placental growth factor (PlGF), were measured. PlGF is important for proper endothelial and vascular function and blood pressure regulation during pregnancy [12–14]. Because PlGF is negatively associated with body weight in pregnant women [15, 16], it was assessed whether PlGF levels were altered in response to HFD in the pregnant offspring groups in this study.

## Materials and methods

### Animals, diet protocols, and timed pregnancies

**Fig 1:**
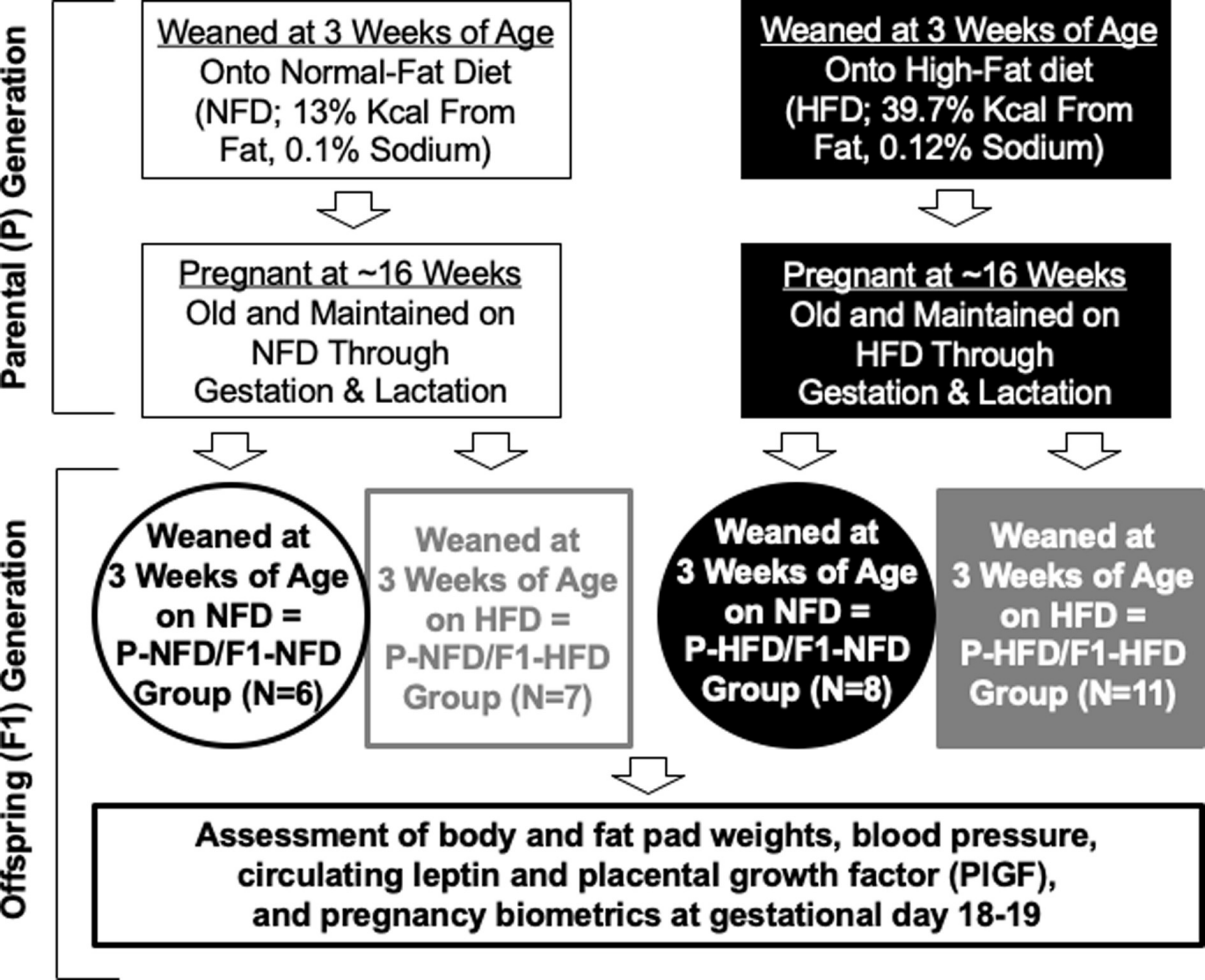
Scheme illustrating the series of events in generating the experimental timed-pregnant rats utilized in this study. At 3 weeks old, Wistar Hannover female rats were started on NFD or HFD, and at ~16 weeks of age, rats were mated with males on Envigo 8640 standard chow. These pregnant females were the parental (P) generation and maintained on respective NFD or HFD throughout pregnancy and lactation during this perinatal period. The F1 generation of offspring were weaned onto either NFD or HFD that were then mated with Wistar Hannover males to generate the following 4 groups of timed-pregnant experimental offspring: 1) P-NFD/F1-NFD (exposed to NFD during perinatal period and from weaning through pregnancy), 2) P-HFD/F1-NFD (exposed to HFD during perinatal period but fed NFD from weaning through pregnancy), 3) P-NFD/F1-HFD (exposed to NFD during perinatal period but fed HFD from weaning through pregnancy), and 4) P-HFD/F1-HFD (exposed to HFD during perinatal period and from weaning through pregnancy).

The Institutional Animal Care and Use Committee approved all animal protocols and rats were housed in AAALAC accredited animal facilities at The University of Mississippi Medical Center. Fig 1 illustrates the study design used for generating experimental rats, which were generated from an in-house colony of Wistar Hannover (WH) rats maintained on Envigo 8640 standard chow. This diet is formulated to support growth and reproduction in rodents and has a crude protein content of 22%. From this colony, female rats were weaned at 3 weeks of age and started on purified diets of either normal-fat diet (NFD; Teklad cat# TD.07055; composed of % kcal from: 13.0% fat, 68.0% carbohydrates, and 19.0% protein with a caloric content of 3.6 kcal/g and 0.1% sodium) or high-fat diet (HFD; Teklad, cat# TD.07054; composed of % kcal from: 39.7% fat, 41.5% carbohydrates, and 18.8% protein with a caloric content of 4.3 kcal/g and 0.12% sodium). Rats were maintained on respective diets until ~16 weeks of age when they were mated with WH males that had been maintained on standard chow. The pregnant rats were maintained on their respective diets through the perinatal period, which includes the timeframe encompassing gestation of the fetus and lactation until their offspring were weaned at 3 weeks of age. These pregnant dams were defined as the parental, P generation and termed P-NFD and P-HFD. There was no difference (P = 0.6) in the number of offspring (first filial, F1 generation) born from P-NFD (11±2) versus P-HFD (12±1) groups. Upon weaning at 3 weeks of age, the female F1 offspring either remained on the same diets as the P generation or changed to the other diet. These offspring were allowed to age to ~13 weeks old then mated with age-matched brothers on standard chow to generate timed-pregnant rats. Pregnancy was confirmed by observing sperm in the vaginal smear, which was indicative of gestational day 0. The rate of generating successful pregnancies was calculated by (number of successful pregnancies/total number of rats attempted to become pregnant)*100. These breeding and diet paradigms generated 4 experimental groups of timed-pregnant offspring: 1) P-NFD/F1-NFD (N = 6), 2) P-HFD/F1-NFD (N = 8), 3) P-NFD/F1-HFD (N = 7), and 4) P-HFD/F1-HFD (N = 11).

### Body and fat weight assessment

In each of the offspring groups, body weight was assessed from weaning at 3 weeks of age until timed pregnancies were generated at ~13 weeks of age. Body weight was also measured at gestational day 0 and 19.

On gestational day 18, total fat and total lean body composition were examined using an Echo-Magnetic Resonance Imaging (MRI)-700 body composition analyzer (Echo Medical Systems, Houston, TX, USA). Rats were placed in restraint cages and the average of two scans recorded for each rat. On day 19, adipose tissues were harvested and wet weights recorded, which included the visceral retroperitoneal adipose tissue surrounding the kidneys [17]; parametrial adipose tissue; and subcutaneous adipose tissue from the interscapular space.

### Food intake

Rats were housed individually and cumulative food intake was assessed between gestational days 0 and 19 in their home cages.

### Blood pressure measurement

On gestational day 18, rats were anesthetized with 2–4% isoflurane then indwelling catheters were implanted in the left carotid artery and exposed at the nape of the neck. Catheters consisted of V/1 tubing attached to V/3 tubing (Scientific Commodities, Lake Havasu City, AZ, USA). Approximately 2.5 cm of the V/3 end of the catheter was inserted into the carotid. Catheters were filled with sterile heparin-0.9% saline solution (300 mg/mL; Pfizer, New York City, NY, USA) and stoppered with a stainless-steel catheter plug (SP22/12; Instech Laboratories, Plymouth Meeting, PA, USA) to maintain patency. Conscious mean arterial blood pressure was measured on the next day at gestational day 19 as described previously [18]. For this purpose, rats were placed in restrainers (Kent Scientific Corp, Torrington, CT, USA) and catheters connected to pressure transducers (MLT0699; ADInstruments, Colorado Springs, CO, USA) coupled to a computerized data acquisition system (PowerLab, ADInstruments). Readings were calibrated on every rat then data acquired at 1k/s. Once blood pressure readings stabilized (~1h), ~10 min of mean arterial blood pressure (MAP) data were collected and averaged.

### Blood collection and pregnancy biometrics

On gestational day 19, rats were anesthetized with 2–4% isoflurane then a midline incision was made and uterine horns with fetuses exteriorized. Blood was collected from the abdominal aorta into Vacutainer K_2_EDTA tubes (BD, Franklin Lakes, NJ, USA), spun at 2500 rpm for 12 min at 4°C, and plasma stored at -20°C. It was ensured that each fetus and matching placenta were weighed and recorded as individual fetal-placental units. Average placental and fetal weights were calculated per rat and then averaged for each experimental group. Placental sufficiency = average viable fetal weight/average placental weight for each dam as a surrogate measure of the nurturing capacity of the placenta, as previously described in humans [19]. Total viable fetuses were noted. Percent fetal resorption = (number of resorbed fetuses/total number of fetuses)*100. Immediately following removal of tissues and blood, all animals were killed under isoflurane anesthesia by thoracotomy and removal of the heart.

### Plasma biochemistry

Plasma levels of the adipokine, leptin, were examined using a Quantikine enzyme-linked immunosorbent assay (ELISA) from R&D Systems (Minneapolis, MN, USA—MOB00). Plasma free PlGF levels were also quantified by an ELISA kit from R&D Systems detecting mouse PlGF-2 (Minneapolis, MN, USA—MP200).

### Statistical analysis

Data were graphed and analyzed using GraphPad Prism version 8.1.2 (La Jolla, CA, USA). Data are presented as mean ± standard error of the mean (SEM). Statistical significance was defined as P<0.05 and determined between all 4 pregnant groups by a two-way ANOVA followed by Tukey’s multiple comparisons tests. A two-way ANOVA with repeated measures followed by a Tukey’s multiple comparisons test was used to assess significance of pre-pregnancy body weights from 3 to 13 weeks of age between each of the 4 groups. Statistical symbols are only presented in the graphs if the two-way ANOVA detected an interaction between the 2 factors of pre-natal (P) versus offspring post-natal (F1) diet groups and the post-hoc test indicated specific differences between any of the 4 groups.

## Results

### Pre-pregnancy body weights

**Fig 2:**
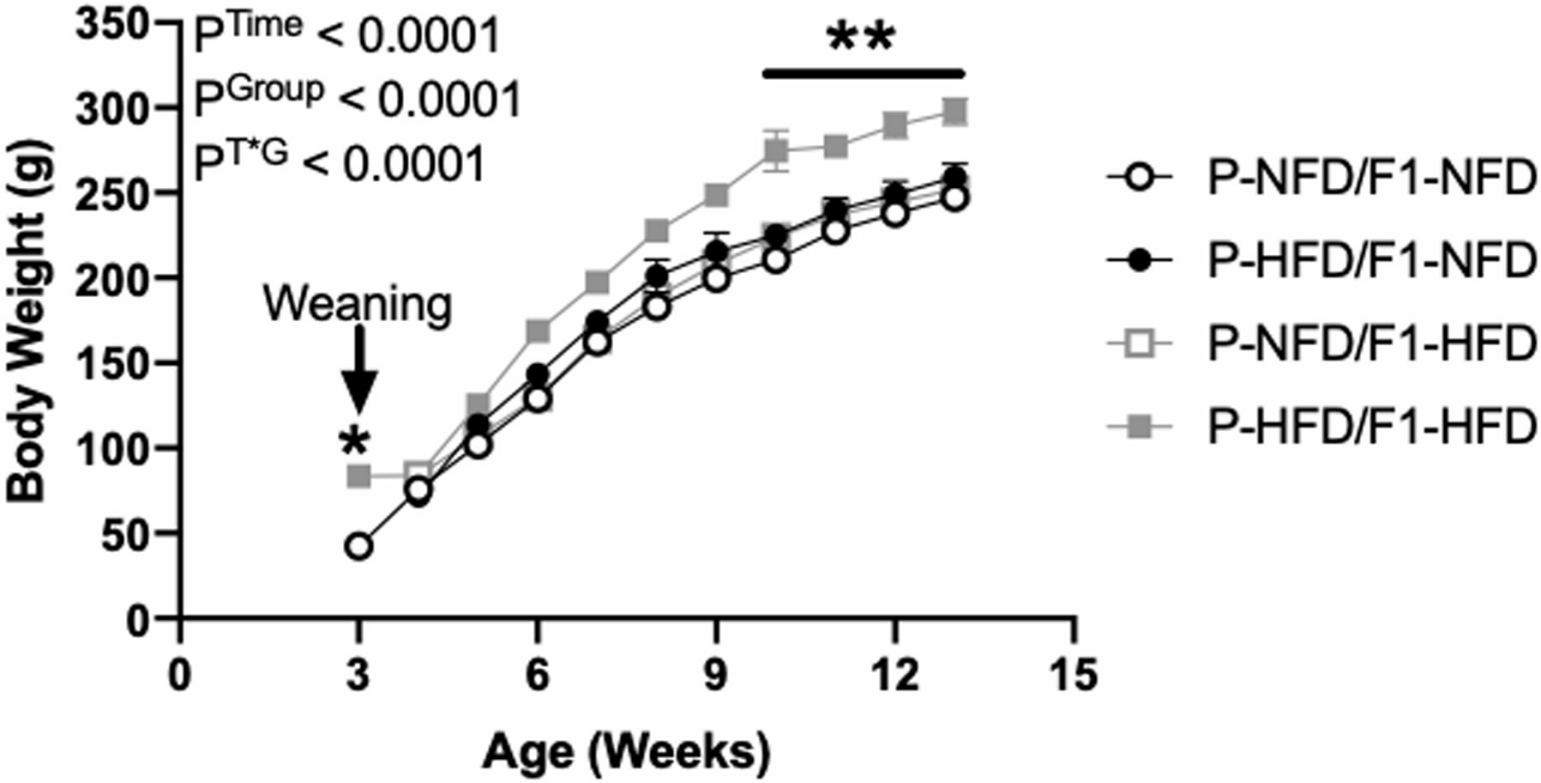
Continuous body weight measurements in non-pregnant P-NFD/F1-NFD, P-HFD/F1-NFD, P-NFD/F1-HFD, and P-HFD/F1-HFD offspring groups. Body weights were assessed from weaning at 3 weeks of age until 13 weeks of age, which was directly before timed pregnancies were generated. Inset are results from the two-way ANOVA. Results from Tukey’s multiple comparisons test: *P<0.0001 for body weight in offspring from P-HFD vs. P-NFD at 3 weeks of age. **P<0.001 for body weight in P-HFD/F1-HFD vs. all other groups from 10–13 weeks of age.

Upon weaning at 3 weeks of age, body weight was significantly greater in the female F1 offspring from P-HFD versus P-NFD dams (Fig 2). Body weight was then tracked weekly until 13 weeks of age in each of the 4 groups of the female F1 offspring: 1) P-NFD/F1-NFD (N = 6), 2) P-HFD/F1-NFD (N = 8), 3) P-NFD/F1-HFD (N = 7), and 4) P-HFD/F1-HFD (N = 11). As Fig 2 illustrates, body weight was numerically greater in the P-HFD/F1-HFD group by 6 weeks of age compared to all other 3 groups. However, their body weight did not reach statistical significance until 10 weeks of age and continued to be greater through 13 weeks of age (Fig 2). Cumulative weight gain from 3 to 13 weeks of age was also greatest (P = 0.002) in the P-HFD/F1-HFD group (242±10 g), as compared to P-NFD/F1-NFD (210±4 g), P-HFD/F1-NFD (202±6 g), and P-NFD/F1-HFD (209±6 g) groups. Overall, pre-pregnancy body weight was greatest in the P-HFD/F1-HFD before timed pregnancies were generated at ~13 weeks old.

### Pregnancy success rates

The number of days (i.e., smears performed) while attempting to generate timed-pregnant rats were not significantly different (P = 0.2) between any of the groups (P-NFD/F1-NFD: 4±1 days, P-HFD/F1-NFD: 10±3 days, P-NFD/F1-HFD: 6±2 days, and P-HFD/F1-HFD: 7±1 days). The rate of generating successful pregnancies was 86% in P-NFD/F1-NFD, 75% in P-HFD/F1-NFD, 100% of rats in P-NFD/F1-HFD, and 71% in the P-HFD/F1-HFD group.

### Food intake during pregnancy

Cumulative food intake from gestational day 0–19 was 320±10 g for P-NFD/F1-NFD, 322±14 for P-HFD/F1-NFD, 267±2 for P-NFD/F1-HFD, and 284±21 for P-HFD/F1-HFD. The two-way ANOVA detected a significant main effect (P = 0.007) in the post-natal F1-HFD vs. F1-NFD diet groups.

### Body and fat weights during pregnancy

**Fig 3:**
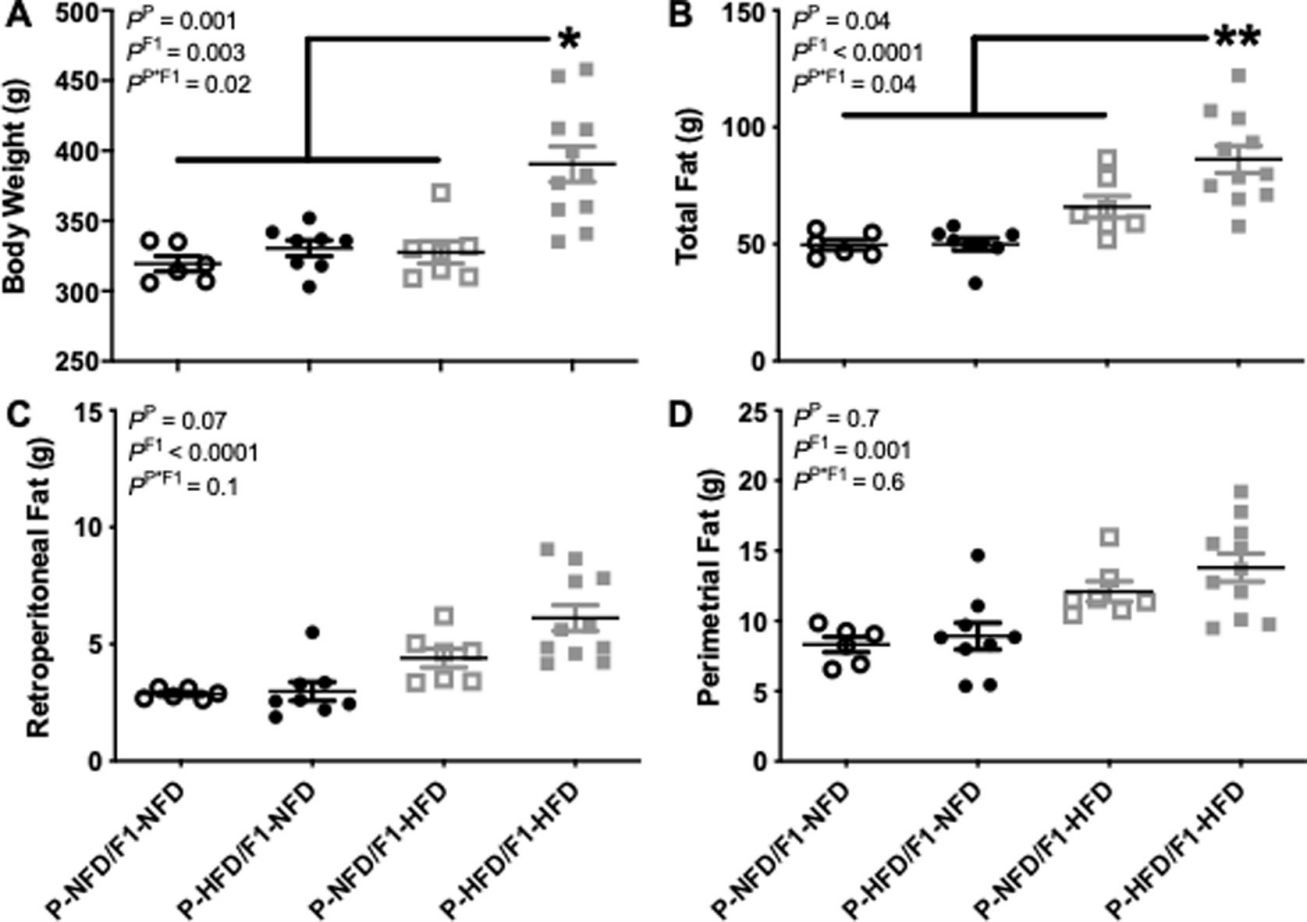
Body and fat weights during pregnancy. Body weight (A), EchoMRI total body fat (B), isolated retroperitoneal fat (C), and parametrial fat (D) in P-NFD/F1-NFD, P-HFD/F1-NFD, P-NFD/F1-HFD, and P-HFD/F1-HFD pregnant groups at gestational day 18–19. Inset are results from the two-way ANOVA. *P = 0.02 and **P = 0.04 for P-HFD/F1-HFD vs. all other groups.

It was examined if HFD feeding during the P generation exaggerated HFD-induced increases in body and fat weights in the pregnant F1 offspring. Gestational weight gain from gestational day 0–19 was comparable (P = 0.2) between all 4 groups: P-NFD/F1-NFD (71±1 g), P-HFD/F1-NFD (71±1 g), P-NFD/F1-HFD (64±8 g), and P-HFD/F1-HFD (75±7 g). At the end of pregnancy, body weight, total body fat, retroperitoneal fat, parametrial fat, and subcutaneous fat were weights were evaluated. Body weight (Fig 3A) and total body fat (Fig 3B) were not different between P-NFD/F1-NFD, P-HFD/F1-NFD, or P-NFD/F1-HFD but were significantly greater in P-HFD/F1-HFD compared to all other pregnant groups. Retroperitoneal fat (Fig 3C) and parametrial fat (Fig 3D) were similar between P-NFD/F1-NFD, P-HFD/F1-NFD, P-NFD/F1-HFD, and P-HFD/F1-HFD pregnant groups; however, the two-way ANOVA detected a main effect for the post-natal F1-HFD vs. F1-NFD diet groups for retroperitoneal (P<0.0001) and parametrial fat (P = 0.001) weights. Subcutaneous fat weights were similar (P = 0.9) among all 4 groups: P-NFD/F1-NFD (0.97±0.08 g), P-HFD/F1-NFD (0.96±0.08 g), P-NFD/F1-HFD (0.97±0.08), and P-HFD/F1-HFD (0.96±0.08 g).

At the end of pregnancy, total body lean mass values were 254±2 g for P-NFD/F1-HFD, 270±3 g for P-HFD/F1-NFD, 248±3 g for P-NFD/F1-HFD, and 283±8 for the P-HFD/F1-HFD pregnant group. The two-way ANOVA detected a significant main effect (P = 0.003) for the pre-natal P-HFD vs. P-NFD diet groups. Tibia lengths were measured in a subset of rats and were similar (P = 0.5) between P-NFD/F1-NFD (3.6±0.03 cm, N = 4), P-HFD/F1-NFD (3.6±0.02 cm, N = 3), P-NFD/F1-HFD (3.6±0.03 cm, N = 6), and P-HFD/F1-HFD (3.7±0.07 cm, N = 3).

### Circulating leptin during pregnancy

**Fig 4:**
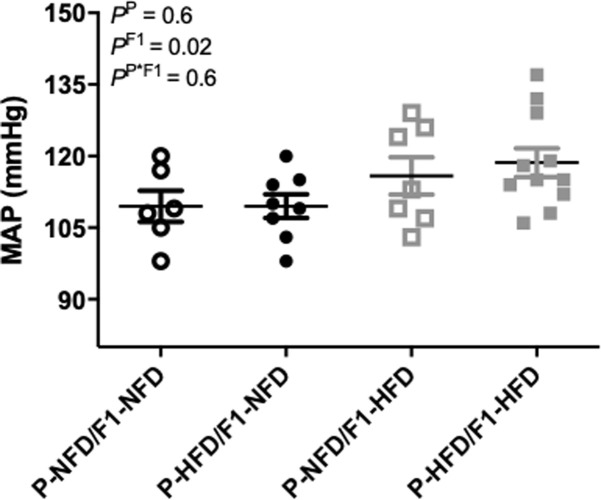
Circulating levels of the adipokine, leptin. Plasma leptin was measured in P-NFD/F1-NFD, P-HFD/F1-NFD, P-NFD/F1-HFD, and P-HFD/F1-HFD pregnant groups at gestational day 19. Inset are results from the two-way ANOVA.

Plasma leptin levels were similar (P = 0.6) among the P-NFD/F1-NFD, P-HFD/F1-HFD, P-NFD/F1-HFD, and P-HFD/F1-HFD pregnant groups on gestational day 19 (Fig 4).

### Blood pressure during pregnancy

**Fig 5:**
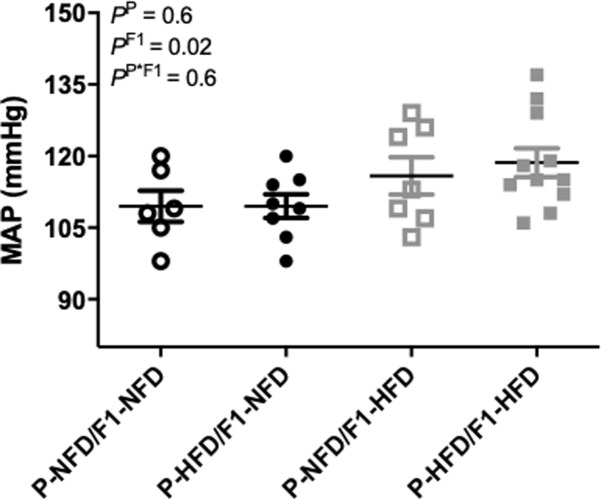
Mean arterial blood pressure (MAP) levels. MAP was measured in P-NFD/F1-NFD, P-HFD/F1-NFD, P-NFD/F1-HFD, and P-HFD/F1-HFD pregnant groups at gestational day 19. Inset are results from the two-way ANOVA.

Conscious mean arterial blood pressure (MAP) was comparable (P = 0.6) between P-NFD/F1-NFD, P-HFD/F1-NFD, P-NFD/F1-HFD, and P-HFD/F1-HFD pregnant offspring groups at gestational day 19 (Fig 5). However, the two-way ANOVA detected a significant main effect (P = 0.02) for post-natal F1-HFD vs. F1-NFD diet groups.

### Circulating PlGF during pregnancy

**Fig 6:**
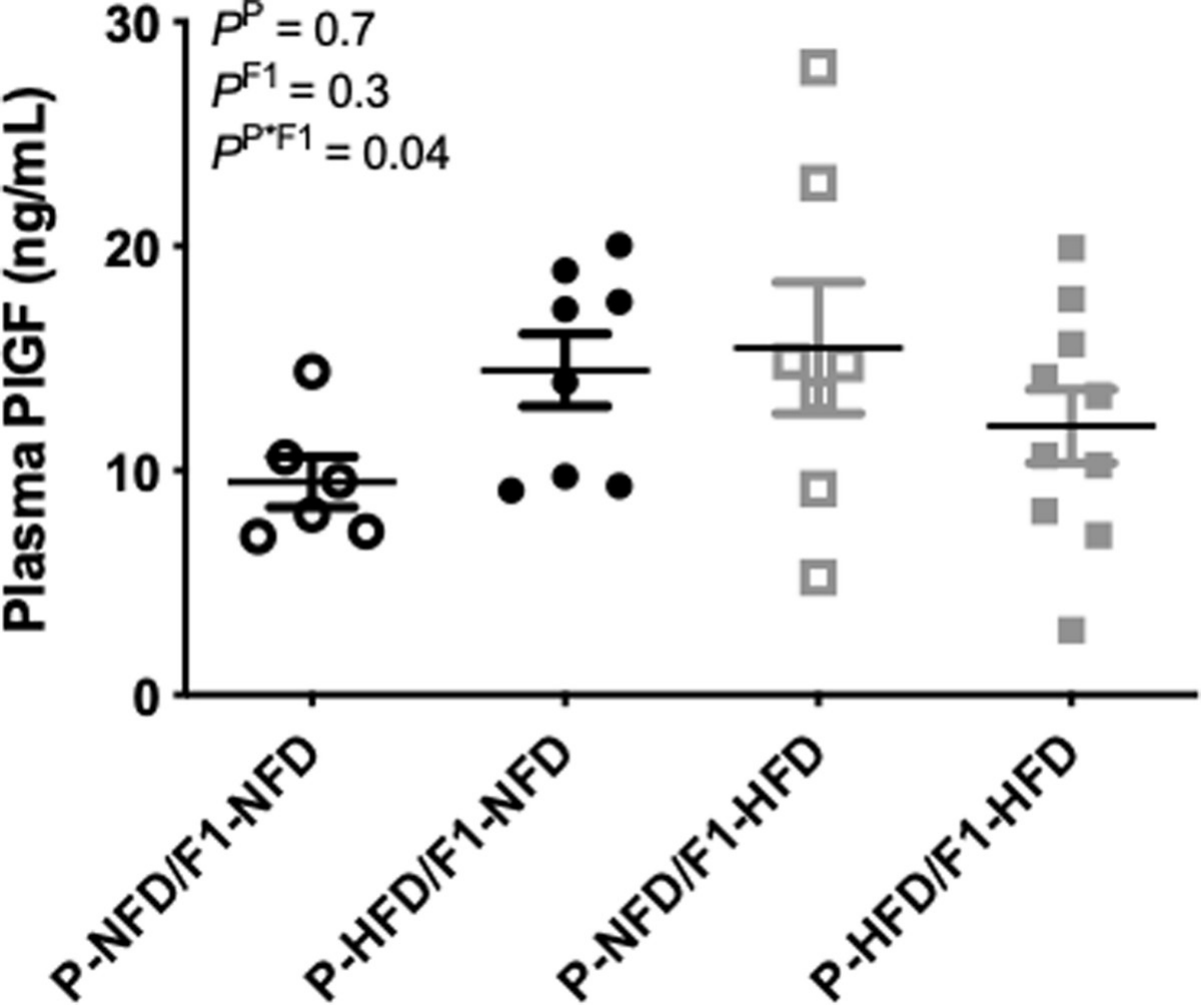
Average circulating placental growth factor (PlGF) levels. Plasma levels of PlGF were measured in P-NFD/F1-NFD, P-HFD/F1-NFD, P-NFD/F1-HFD, and P-HFD/F1-HFD pregnant groups at gestational day 19. Inset are results from the two-way ANOVA.

**Fig 7:**
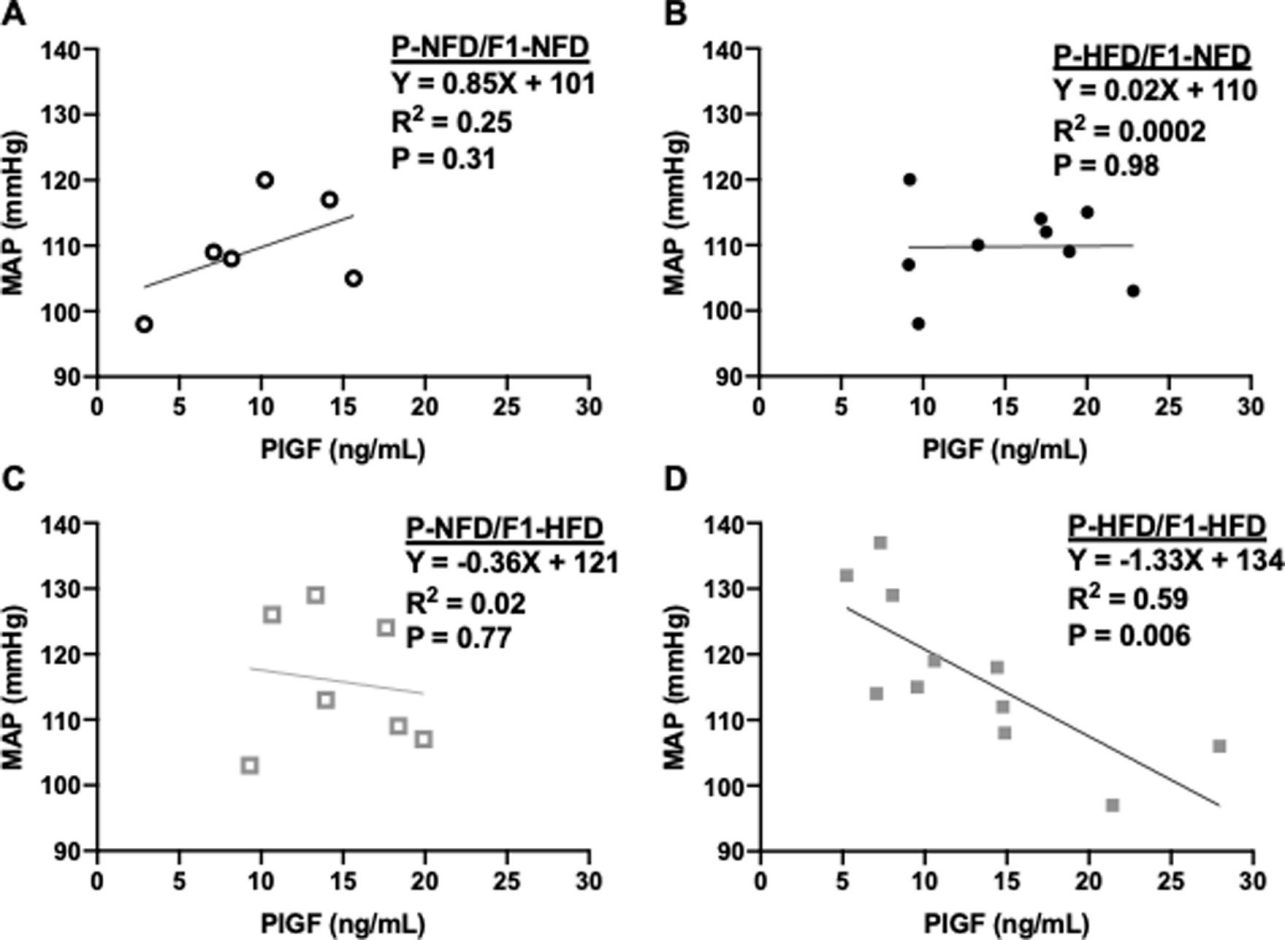
Correlations between plasma placental growth factor (PlGF) levels and mean arterial blood pressure (MAP). Correlations were calculated in P-NFD/F1-NFD (A), P-HFD/F1-NFD (B), P-NFD/F1-HFD (C), and P-HFD/F1-HFD (D) pregnant groups at gestational day 19. Inset are the results for the linear regression analyses and the correlative statistics. Significance was only detected in the P-HFD/F1-HFD group.

Regarding average values for plasma concentrations of PlGF on gestational day 19, the two-way ANOVA detected an interaction (P = 0.04) between the 2 factors of pre-natal (P) versus offspring post-natal (F1) diet groups; however, the post-hoc analysis did not reveal any specific differences between the P-NFD/F1-NFD, P-HFD/F1-HFD, P-NFD/F1-HFD, and P-HFD/F1-HFD pregnant groups (Fig 6). It was observed that PlGF levels were quite variable. Therefore, it was assessed whether there were any correlations between PlGF and blood pressure levels. PlGF is important for proper blood pressure regulation during pregnancy [12]. There were no significant correlations detected between circulating PlGF and blood pressure levels in P-NFD/F1-NFD (Fig 7A), P-HFD/F1-NFD (Fig 7B), or P-NFD/F1-HFD (Fig 7C) pregnant groups. In contrast, there was a significant correlation between these 2 variables in the P-HFD/F1-HFD pregnant group (Fig 7D), whereby those that had the lower levels of PlGF had higher blood pressure levels.

### Pregnancy biometrics

**Fig 8:**
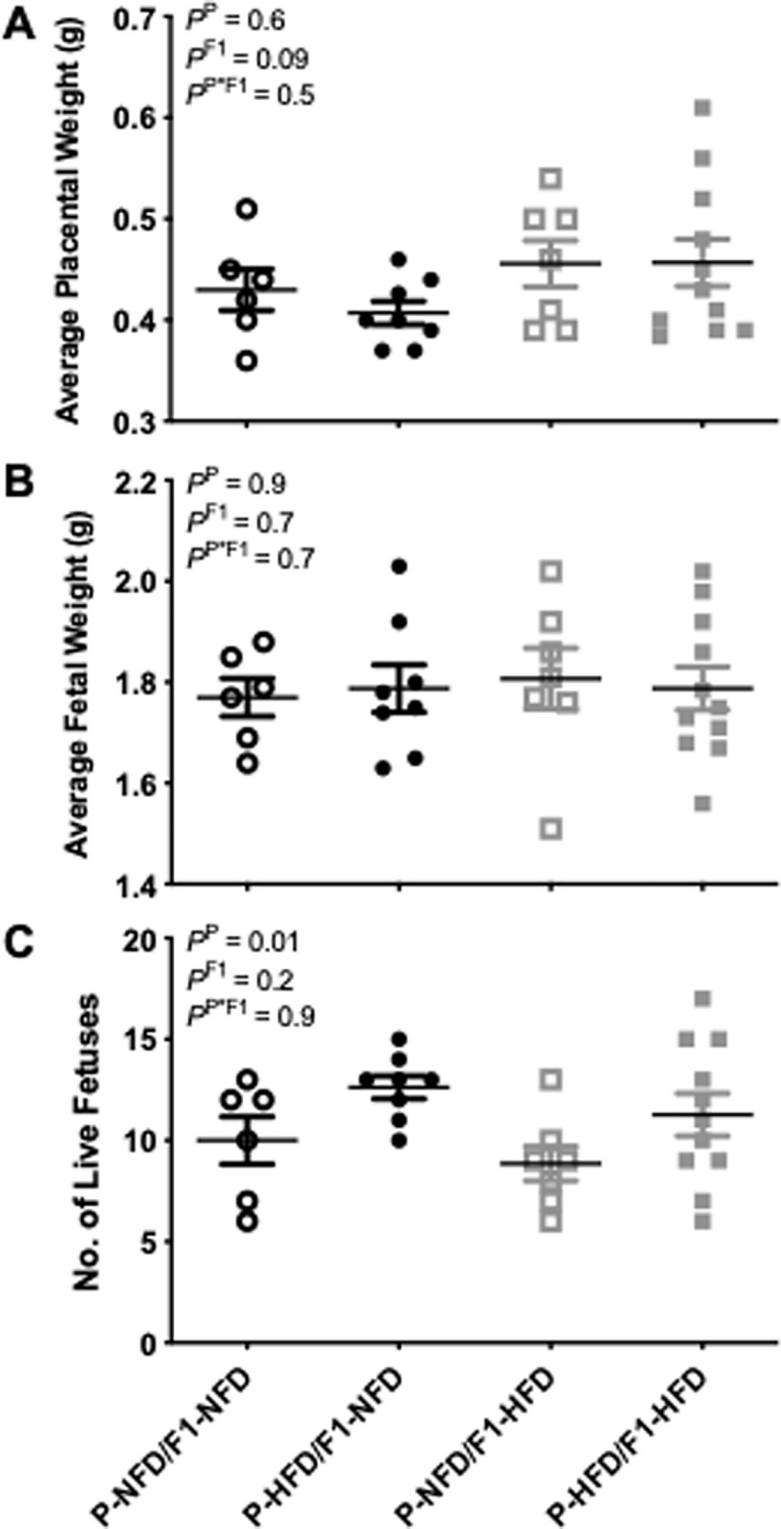
Pregnancy biometrics. Average placenta weight (A), average fetal weight (B), and number of liver fetuses (C) in P-NFD/F1-NFD, P-HFD/F1-NFD, P-NFD/F1-HFD, and P-HFD/F1-HFD pregnant groups at gestational day 19. Inset are results from the two-way ANOVA.

Pregnancy biometrics were measured on gestational day 19. Average placental weights (Fig 8A) and average fetal weights (Fig 8B) were similar (P = 0.5 and P = 0.7, respectively) between all 4 groups of P-NFD/F1-NFD, P-HFD/F1-NFD, P-NFD/F1-HFD, and P-HFD/F1-HFD pregnant rats. Placental sufficiency was comparable (P = 0.4) in P-NFD/F1-NFD (0.43±0.02), P-HFD/F1-NFD (0.41±0.01), P-NFD/F1-HFD (0.46±0.02), and P-HFD/F1-HFD (0.46±0.03) groups. The number of live fetuses was similar (P = 0.9) among all 4 pregnant groups (Fig 8C). Fetal absorption rates were comparable (P = 0.8) in P-NFD/F1-NFD (23±8%), P-HFD/F1-NFD (8±3%), P-NFD/F1-HFD (27±7%), and P-HFD/F1-HFD (17±4%) pregnant groups.

## Discussion

In this study, HFD consumption in the P generation consisted of feeding from weaning and continued through gestation and lactation. Their F1 offspring either remained on the same diet as the P generation or were changed to the other diet. This diet paradigm resulted in 4 groups of experimental F1 offspring: P-NFD/F1-NFD, P-HFD/F1-NFD, P-NFD/F1-HFD, and P-HFD/F1-HFD. Before generating timed-pregnancies, it was noted that the P-HFD/F1-NFD were heavier than any other diet group in the non-pregnant state. Timed-pregnant rats were then generated with pregnancy outcomes assessed at the end of pregnancy between gestational days 18 and 19. Body weight, total body fat mass, and visceral fat weight were similar during pregnancy among the P-NFD/F1-NFD, P-HFD/F1-NFD, and P-NFD/F1-HFD groups. The novel finding was that combined HFD during the P and F1 generations exaggerated obesity and fat mass in the P-HFD/F1-HFD group compared to all other groups.

Circulating levels of the adipokine, leptin, were assessed in this study. Leptin has been shown to be increased in obese patients [20] and to mediate the development of obesity hypertension in studies using non-pregnant rodents; this occurs via downstream signaling of the melanocortin-4 receptor (MC4R) in the brain that promotes sympathetic drive and hypertension [21]. Others have found that MC4R signaling mediates hypertension in those non-pregnant female offspring derived under maternal obesity [22]. However, in this study, leptin and blood pressure levels were similar between all 4 pregnant groups of P-NFD/F1-NFD, P-HFD/F1-NFD, P-NFD/F1-HFD, and P-HFD/F1-HFD rats. Thus, we did not detect increased blood pressure in the pregnant offspring borne from dams on HFD (namely, the P-HFD/F1-NFD and P-HFD/F1-NFD groups), like was shown in other studies using non-pregnant female offspring. We currently do not know the mechanisms whereby these pregnant rats, especially the P-HFD/F1-HFD that had increased body fat, do not have increased blood pressure levels beyond the finding that leptin levels were not altered.

Although there were no differences in the average blood pressure between all 4 groups of pregnant offspring, some of the highest individual blood pressure values were found in the P-HFD/F1-HFD pregnant group of F1 offspring. Recall that the P-HFD/F1-HFD group also had some of the highest body fat weights detected in this study. Human studies have shown a negative association of body weight and circulating levels of the anti-hypertensive factor, PlGF [15, 16]. As PlGF is critically important for proper blood pressure regulation in pregnancy, correlative statistics were performed to assess whether PlGF concentrations were associated with blood pressure levels. Although average PlGF levels were similar between all groups, PlGF was correlated with blood pressure only in the P-HFD/F1-HFD pregnant group. This correlation suggested that blood pressure regulation in the P-HFD/F1-HFD group is sensitive to changes in circulating PlGF levels during pregnancy.

It has been noted that human pregnancies complicated by the hypertensive disorder of preeclampsia have placental ischemia and hypoxia [23]. Preeclamptic patients and experimental animal models of placental ischemia in non-human primates and rodents have increased blood pressure and reduced circulating PlGF levels [13, 24, 25]. When recombinant human PlGF is administered into rats with placental ischemia resulting from reduced uterine perfusion pressure (RUPP), it lowers their blood pressure and restores the expression of endothelial NOS (NOS3) in aortic tissue [12, 26]. PlGF mediates vasorelaxation via activation of NOS in the endothelium, which has been demonstrated in pressurized small mesenteric arteries isolated from late-pregnant rats [14]. Based on the above findings, it would be predicted that the P-HFD/F1-HFD group would have a greater blood pressure response to insults that reduce PlGF; this includes placental ischemia-induced hypertension. We propose that a greater dependence on PlGF during pregnancy in the P-HFD/F1-HFD group may predispose to greater placental ischemia-induced hypertension and vascular dysfunction following the RUPP procedure. Future experiments will assess this proposal using methods to continually measure blood pressure throughout pregnancy. The pregnant P-HFD/F1-HFD model could be useful to study the mechanisms underlying the observation that obesity increases the risk for preeclampsia [27].

A limitation of the current experimental design was that blood pressure levels were not examined prior to pregnancy. As it has been demonstrated that non-pregnant F1 offspring reared from the P-HFD generation have increased blood pressure [1], it could be that we did not find a significant difference between any of our 4 pregnant groups because pregnancy masked non-pregnant raised blood pressure. Upcoming research will track blood pressure before and throughout pregnancy in this novel HFD model. Moreover, future experiments should examine the mechanisms within the adipose tissue that mediate increased HFD-induced adiposity in the P-HFD/F1-HFD pregnant group, a candidate is adrenergic receptor (AR) signaling. There is reduced β3-AR mRNA expression in adipose tissue in non-pregnant offspring derived from diet-induced obese dams [28]. It was learned from β3-AR knockout mice that reductions in this signaling pathway increases the susceptibility to HFD-induced obesity [29]. It has also been demonstrated in humans that activation of β-AR signaling significantly reduces body weight and fat [30]. It would be interesting to examine if activating this receptor system before and/or during pregnancy would attenuate the exaggerated body weight and adipose tissue amounts during P-HFD/F1-HFD pregnancies.

## Conclusions

The present study examined the generational impact of HFD feeding on pregnancy outcomes. It is known that obesogenic conditions like adverse diets increase the risk for altered cardio-metabolic function during pregnancy in the mother and her offspring. Previous studies in these offspring have mainly focused on the non-pregnant state. Studies like the present one in rats and observations in humans indicate that environmental and social stressors in prior generations increase the vulnerability for adverse environments in their offspring [31, 32]. Our experiments are some of the first, to our knowledge, that explored the generational effect of HFD in the P and F1 generations on pregnancy outcomes in the F1 offspring using animal models. Such studies might provide rationale for clinicians to ascertain a woman’s metabolic status not only during her own pregnancy but previous pregnancies in her family, which could direct dietary and/or exercise regimens to prevent complications associated with increasing obesity in pregnant women [33–35].

## References

[1] Desai M, Jellyman JK, Han G, Beall M, Lane RH, Ross MG. Maternal obesity and high-fat diet program offspring metabolic syndrome. Am J Obstet Gynecol. 2014;211(3):237 e1–e13. 10.1016/j.ajog.2014.03.025 24631702 PMC4149836

[2] Masuyama H, Hiramatsu Y. Additive effects of maternal high fat diet during lactation on mouse offspring. PLoS One. 2014;9(3):e92805 10.1371/journal.pone.0092805 24664181 PMC3963955

[3] Fu Q, Olson P, Rasmussen D, Keith B, Williamson M, Zhang KK, et al A short-term transition from a high-fat diet to a normal-fat diet before pregnancy exacerbates female mouse offspring obesity. Int J Obes (Lond). 2016;40(4):564–72. Epub 2015/11/27. 10.1038/ijo.2015.236 26607040 PMC4821711

[4] Khan I, Dekou V, Hanson M, Poston L, Taylor P. Predictive adaptive responses to maternal high-fat diet prevent endothelial dysfunction but not hypertension in adult rat offspring. Circulation. 2004;110(9):1097–102. Epub 2004/08/25. 10.1161/01.CIR.0000139843.05436.A0 . 15326063

[5] Spradley FT, Palei AC, Granger JP. Differential body weight, blood pressure and placental inflammatory responses to normal versus high-fat diet in melanocortin-4 receptor-deficient pregnant rats. J Hypertens. 2016;34(10):1998–2007. 10.1097/HJH.0000000000001059 27467764 PMC5310251

[6] Ahmed H, Hannan JL, Apolzan JW, Osikoya O, Cushen SC, Romero SA, et al A free-choice high-fat, high-sucrose diet induces hyperphagia, obesity, and cardiovascular dysfunction in female cycling and pregnant rats. Am J Physiol Regul Integr Comp Physiol. 2019;316(5):R472–R85. 10.1152/ajpregu.00391.2018 30758976 PMC6589604

[7] Crew RC, Waddell BJ, Mark PJ. Maternal obesity induced by a 'cafeteria' diet in the rat does not increase inflammation in maternal, placental or fetal tissues in late gestation. Placenta. 2016;39:33–40. 10.1016/j.placenta.2016.01.002 . 26992672

[8] Thompson JA, Hardigan TA, Carrillo-Sepulveda MA, Mintz JD, Ergul A, Diamond MP, et al The contribution of Toll-like receptors to placental inflammation in diet-induced maternal obesity. Placenta. 2015;36(10):1204–6. 10.1016/j.placenta.2015.07.132 . 26282853

[9] Hayes EK, Lechowicz A, Petrik JJ, Storozhuk Y, Paez-Parent S, Dai Q, et al Adverse fetal and neonatal outcomes associated with a life-long high fat diet: role of altered development of the placental vasculature. PLoS One. 2012;7(3):e33370 10.1371/journal.pone.0033370 22442686 PMC3307735

[10] Chen C, Xu X, Yan Y. Estimated global overweight and obesity burden in pregnant women based on panel data model. PLoS One. 2018;13(8):e0202183 10.1371/journal.pone.0202183 30092099 PMC6084991

[11] Heslehurst N, Vieira R, Akhter Z, Bailey H, Slack E, Ngongalah L, et al The association between maternal body mass index and child obesity: A systematic review and meta-analysis. PLoS Med. 2019;16(6):e1002817 10.1371/journal.pmed.1002817 31185012 PMC6559702

[12] Spradley FT, Tan AY, Joo WS, Daniels G, Kussie P, Karumanchi SA, et al Placental Growth Factor Administration Abolishes Placental Ischemia-Induced Hypertension. Hypertension. 2016;67(4):740–7. 10.1161/HYPERTENSIONAHA.115.06783 26831193 PMC4786447

[13] Karumanchi SA. Angiogenic Factors in Preeclampsia: From Diagnosis to Therapy. Hypertension. 2016;67(6):1072–9. 10.1161/HYPERTENSIONAHA.116.06421 . 27067718

[14] Mandala M, Gokina N, Barron C, Osol G. Endothelial-derived hyperpolarization factor (EDHF) contributes to PlGF-induced dilation of mesenteric resistance arteries from pregnant rats. J Vasc Res. 2012;49(1):43–9. 10.1159/000329821 21985802 PMC3221268

[15] Ghosh SK, Raheja S, Tuli A, Raghunandan C, Agarwal S. Serum placental growth factor as a predictor of early onset preeclampsia in overweight/obese pregnant women. J Am Soc Hypertens. 2013;7(2):137–48. 10.1016/j.jash.2012.12.006 . 23394804

[16] Jaaskelainen T, Heinonen S, Hamalainen E, Pulkki K, Romppanen J, Laivuori H, et al Impact of obesity on angiogenic and inflammatory markers in the Finnish Genetics of Pre-eclampsia Consortium (FINNPEC) cohort. Int J Obes (Lond). 2019;43(5):1070–81. 10.1038/s41366-018-0217-8 . 30254363

[17] Allan PLP, Baxter GM, Weston MJ. Clinical ultrasound. 3rd ed. Edinburgh: Churchill Livingstone; 2011.

[18] Spradley FT, Palei AC, Granger JP. Obese melanocortin-4 receptor-deficient rats exhibit augmented angiogenic balance and vasorelaxation during pregnancy. Physiol Rep. 2013;1(4):e00081 10.1002/phy2.81 24159378 PMC3804345

[19] Hunt K, Kennedy SH, Vatish M. Definitions and reporting of placental insufficiency in biomedical journals: a review of the literature. Eur J Obstet Gynecol Reprod Biol. 2016;205:146–9. 10.1016/j.ejogrb.2016.08.029 . 27591716

[20] van Dielen FM, van't Veer C, Schols AM, Soeters PB, Buurman WA, Greve JW. Increased leptin concentrations correlate with increased concentrations of inflammatory markers in morbidly obese individuals. Int J Obes Relat Metab Disord. 2001;25(12):1759–66. Epub 2002/01/10. 10.1038/sj.ijo.0801825 . 11781755

[21] da Silva AA, do Carmo JM, Hall JE. Role of leptin and central nervous system melanocortins in obesity hypertension. Curr Opin Nephrol Hypertens. 2013;22(2):135–40. 10.1097/MNH.0b013e32835d0c05 23299052 PMC3905446

[22] Samuelsson AS, Mullier A, Maicas N, Oosterhuis NR, Eun Bae S, Novoselova TV, et al Central role for melanocortin-4 receptors in offspring hypertension arising from maternal obesity. Proc Natl Acad Sci U S A. 2016;113(43):12298–303. 10.1073/pnas.1607464113 27791019 PMC5087049

[23] Espinoza J. Uteroplacental ischemia in early- and late-onset pre-eclampsia: a role for the fetus? Ultrasound Obstet Gynecol. 2012;40(4):373–82. Epub 2012/11/20. 10.1002/uog.12280 . 23161443

[24] Spradley FT, Ge Y, Haynes BP, Granger JP, Anderson CD. Adrenergic receptor blockade attenuates placental ischemia-induced hypertension. Physiol Rep. 2018;6(17):e13814 Epub 2018/09/20. 10.14814/phy2.13814 30229567 PMC6121121

[25] Makris A, Yeung KR, Lim SM, Sunderland N, Heffernan S, Thompson JF, et al Placental Growth Factor Reduces Blood Pressure in a Uteroplacental Ischemia Model of Preeclampsia in Nonhuman Primates. Hypertension. 2016;67(6):1263–72. 10.1161/HYPERTENSIONAHA.116.07286 27091894 PMC4867111

[26] Zhu M, Ren Z, Possomato-Vieira JS, Khalil RA. Restoring placental growth factor-soluble fms-like tyrosine kinase-1 balance reverses vascular hyper-reactivity and hypertension in pregnancy. Am J Physiol Regul Integr Comp Physiol. 2016;311(3):R505–21. 10.1152/ajpregu.00137.2016 27280428 PMC5142222

[27] Mrema D, Lie RT, Ostbye T, Mahande MJ, Daltveit AK. The association between pre pregnancy body mass index and risk of preeclampsia: a registry based study from Tanzania. BMC Pregnancy Childbirth. 2018;18(1):56 Epub 2018/02/23. 10.1186/s12884-018-1687-3 29466949 PMC5822591

[28] Samuelsson AM, Matthews PA, Argenton M, Christie MR, McConnell JM, Jansen EH, et al Diet-induced obesity in female mice leads to offspring hyperphagia, adiposity, hypertension, and insulin resistance: a novel murine model of developmental programming. Hypertension. 2008;51(2):383–92. 10.1161/HYPERTENSIONAHA.107.101477 . 18086952

[29] Preite NZ, Nascimento BP, Muller CR, Americo AL, Higa TS, Evangelista FS, et al Disruption of beta3 adrenergic receptor increases susceptibility to DIO in mouse. J Endocrinol. 2016;231(3):259–69. 10.1530/JOE-16-0199 27672060 PMC5609459

[30] Bogacka I, Gettys TW, de Jonge L, Nguyen T, Smith JM, Xie H, et al The effect of beta-adrenergic and peroxisome proliferator-activated receptor-gamma stimulation on target genes related to lipid metabolism in human subcutaneous adipose tissue. Diabetes Care. 2007;30(5):1179–86. 10.2337/dc06-1962 . 17351280

[31] Wallack L, Thornburg K. Developmental Origins, Epigenetics, and Equity: Moving Upstream. Matern Child Health J. 2016;20(5):935–40. 10.1007/s10995-016-1970-8 . 27029539

[32] Messer LC, Boone-Heinonen J, Mponwane L, Wallack L, Thornburg KL. Developmental Programming: Priming Disease Susceptibility for Subsequent Generations. Curr Epidemiol Rep. 2015;2(1):37–51. 10.1007/s40471-014-0033-1 26366336 PMC4563822

[33] Mbah AK, Kornosky JL, Kristensen S, August EM, Alio AP, Marty PJ, et al Super-obesity and risk for early and late pre-eclampsia. BJOG. 2010;117(8):997–1004. 10.1111/j.1471-0528.2010.02593.x . 20482533

[34] Ovalle A, Martinez MA, Fuentes A, Marques X, Vargas F, Vergara P, et al [Obesity, a risk factor for ascending bacterial infection during pregnancy]. Rev Med Chil. 2016;144(4):476–82. 10.4067/S0034-98872016000400008 . 27401379

[35] Johansson S, Villamor E, Altman M, Bonamy AK, Granath F, Cnattingius S. Maternal overweight and obesity in early pregnancy and risk of infant mortality: a population based cohort study in Sweden. BMJ. 2014;349:g6572 10.1136/bmj.g6572 25467170 PMC4252825

