# Correction: High-fat diet from parental generation exaggerates body and adipose tissue weights in pregnant offspring

**DOI:** 10.1371/journal.pone.0249968

**Published:** 2021-04-06

**Authors:** The PLOS ONE Staff

Fig 4 is incorrect. The publisher apologizes for the error. The author has provided a corrected version here.

**Fig 4:**
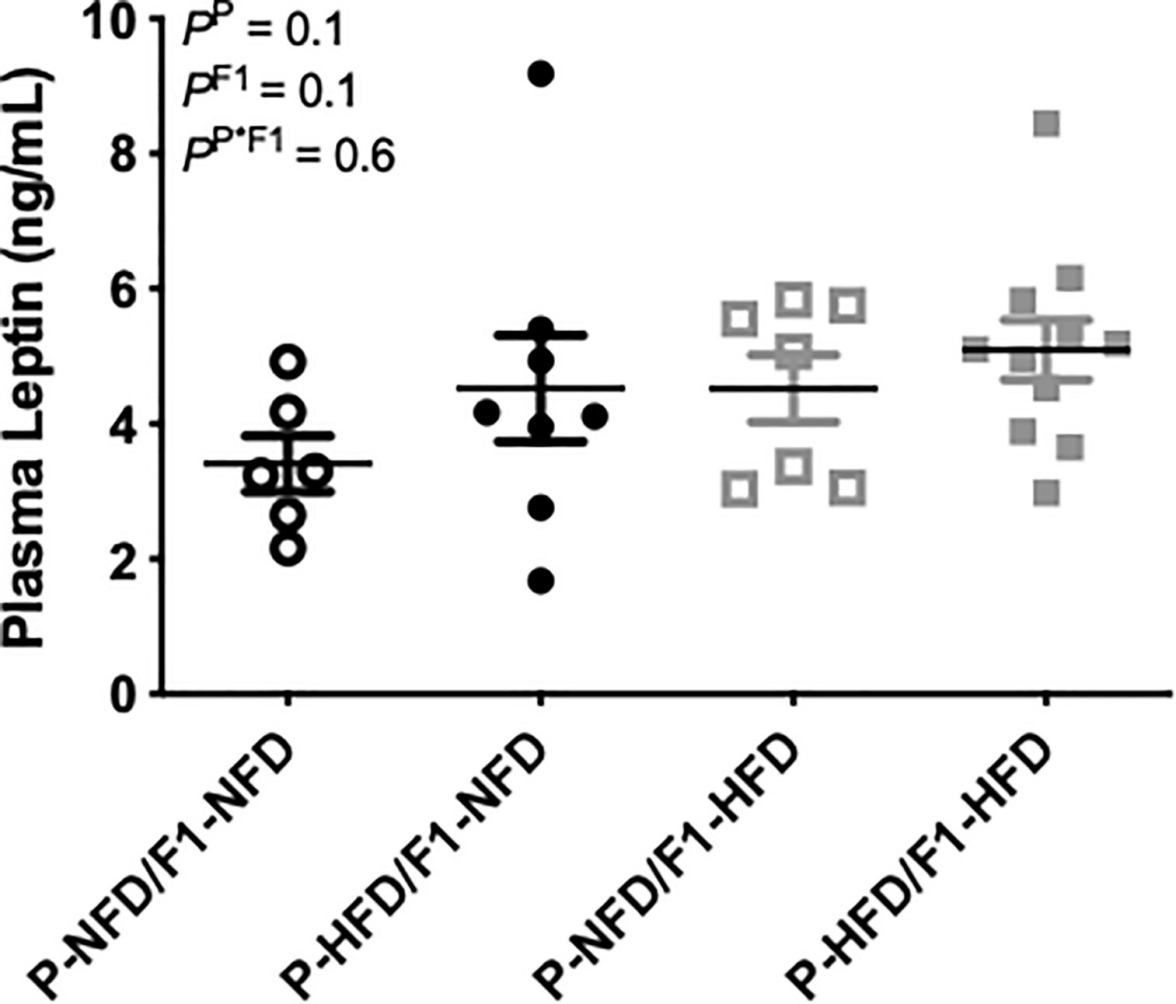
Circulating levels of the adipokine, leptin. Plasma leptin was measured in P-NFD/F1-NFD, P-HFD/F1-NFD, P-NFD/F1-HFD, and P-HFD/F1-HFD pregnant groups at gestational day 19. Inset are results from the two-way ANOVA.
